# Supplementary material for: A second open reading frame in human enterovirus determines viral replication in intestinal epithelial cells
Source: Nat Commun. 2019 Sep 6;10:4066. doi: 10.1038/s41467-019-12040-9 (PMC6731315; doi:10.1038/s41467-019-12040-9)
Supplement: Supplementary file 1 — Supplementary Information [file 41467_2019_12040_MOESM1_ESM.pdf]

Supplementary Information

A second open reading frame in human enterovirus determines viral replication in intestinal epithelial cells

Guo et. al

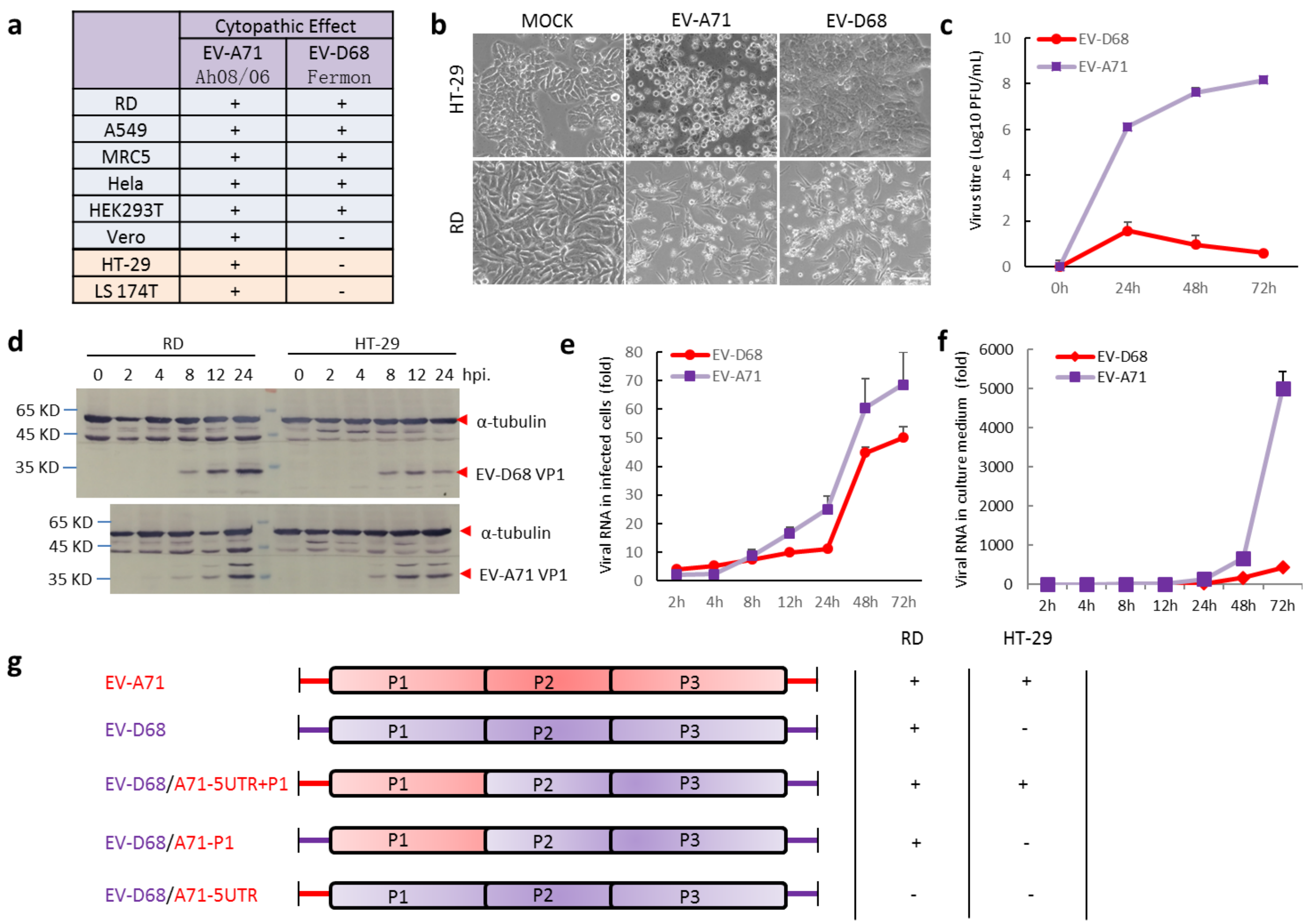

Supplementary Figure 1. The replication capacity of EV-D68 is less efficient than that of EV-A71 in human intestinal HT-29 cells. a, Induction of the cytopathic effect (CPE) by EV-A71 and EV-D68 in various cell lines. “+” indicates more than 50% cell death in virus-infected cultures; “-” indicates no observed cell death in virus-infected cultures compared to that in uninfected cultures. b, RD and HT-29 cells were infected with EV-A71 or EV-D68 at an MOI of 0.1, and the cells were imaged via light microscopy at 72 hpi. Scale bars equal 50 μm. c, Viral titres in the supernatants of the treated cells as described in b were determined at 0, 24, 48, and 72 hpi (n = 3 biologically independent experiments). Error bars denote SEM. d, HT-29 cells were challenged with EV-A71 or EV-D68 at an MOI of 0.1. At 0, 2, 4, 8, 12, and 24 hpi, EV-A71 (top panel)- and EV-D68 (bottom panel)-infected cells were harvested for immunoblotting using the indicated antibodies. e,f, The cells and supernatants of the cells described in panel d were also collected and prepared for RT-PCR assays to detect intracellular EV-D68/EV-A71 RNA (e) (n = 2 biologically independent experiments) and viral RNA in the culture medium (f) (n = 3 biologically independent experiments). Error bars denote SEM. g, Schematic representation of the artificially engineered chimeric EV-A71/EV-D68 viruses. HT-29 cells were challenged with equal amounts of the indicated viruses. “+” indicates more than 50% cell death in virus-infected cultures; “-” indicates no observed cell death in virus-infected cultures compared to that in uninfected cultures.

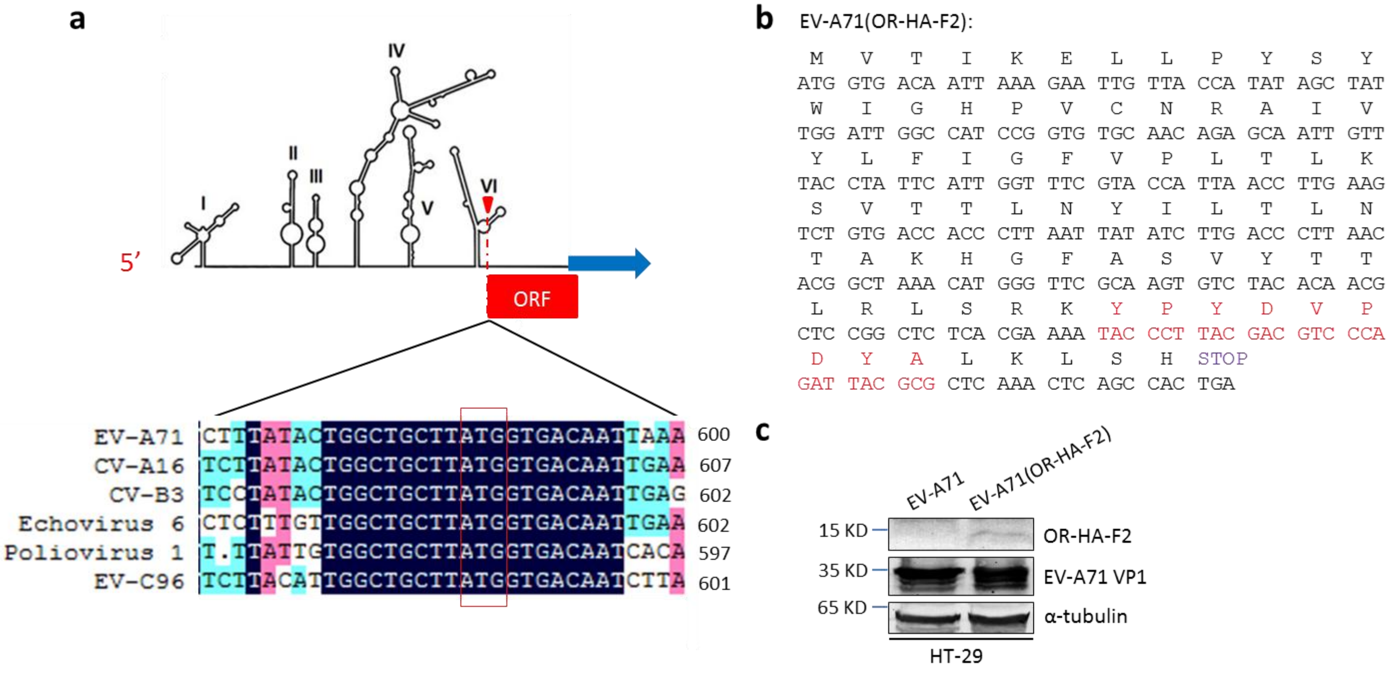

Supplementary Figure 2. A second ORF in EV-A71. a, An alternative initial codon at the 3′ border of the 5’UTR is highly conserved among enteric enteroviruses. b, Amino acid and nucleic acid sequences of ORF2p with HA-Tag (YPYDVPDYA) insertion. c, HT-29 cells were transfected with RNA transcripts of EV-A71 or EV-A71 (OR-HA-F2), and the cells were harvested after 48 h and prepared for immunoblotting using the indicated antibodies.

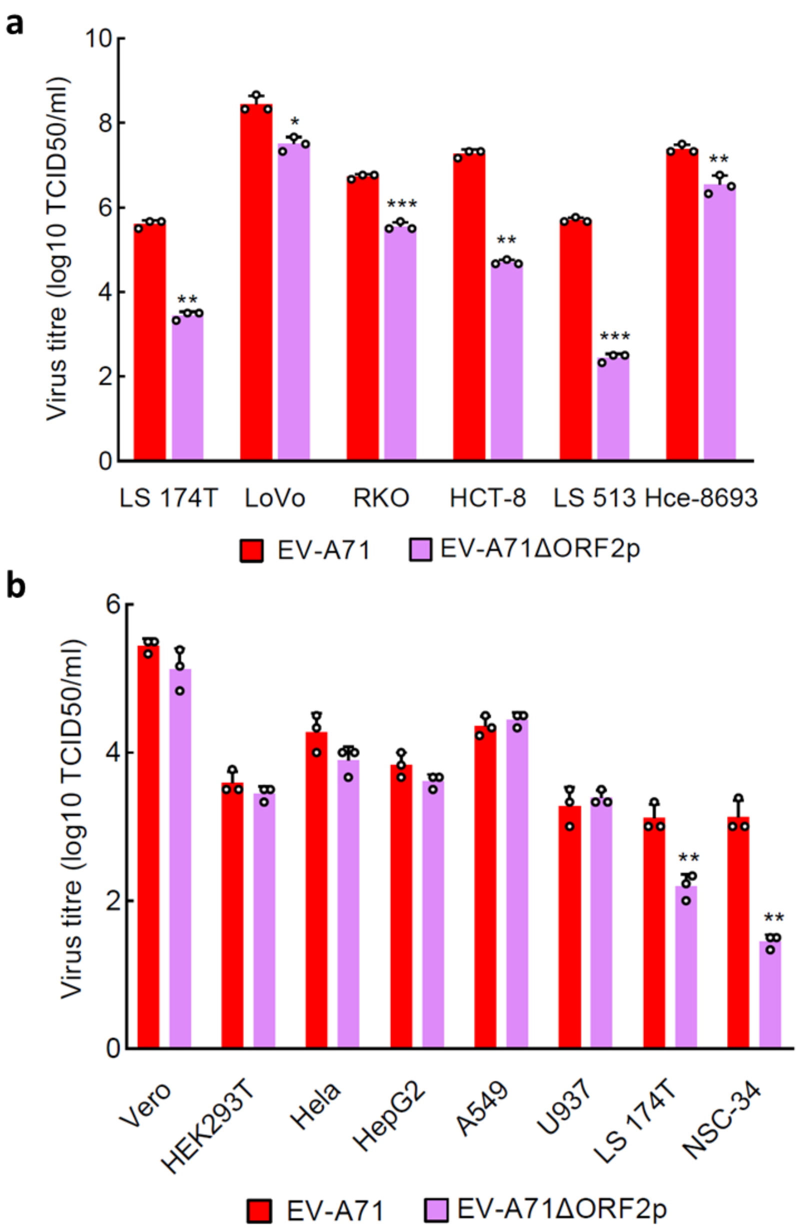

Supplementary Figure 3. ORF2p is essential for EV-A71 replication in intestinal cell lines. a, The indicated cells were infected with EV-A71 or EV-A71ΔORF2p at an MOI of 10. Viral titres were determined at 12 hpi. b, Indicated cells were infected with EV-A71 or EV-A71ΔORF2p at an MOI of 1. Viral titres were determined at 72 hpi. Error bars denote SEM; ANOVA test, n = 3 biologically independent experiments; *p < 0.05, **p < 0.01, ***p < 0.001.

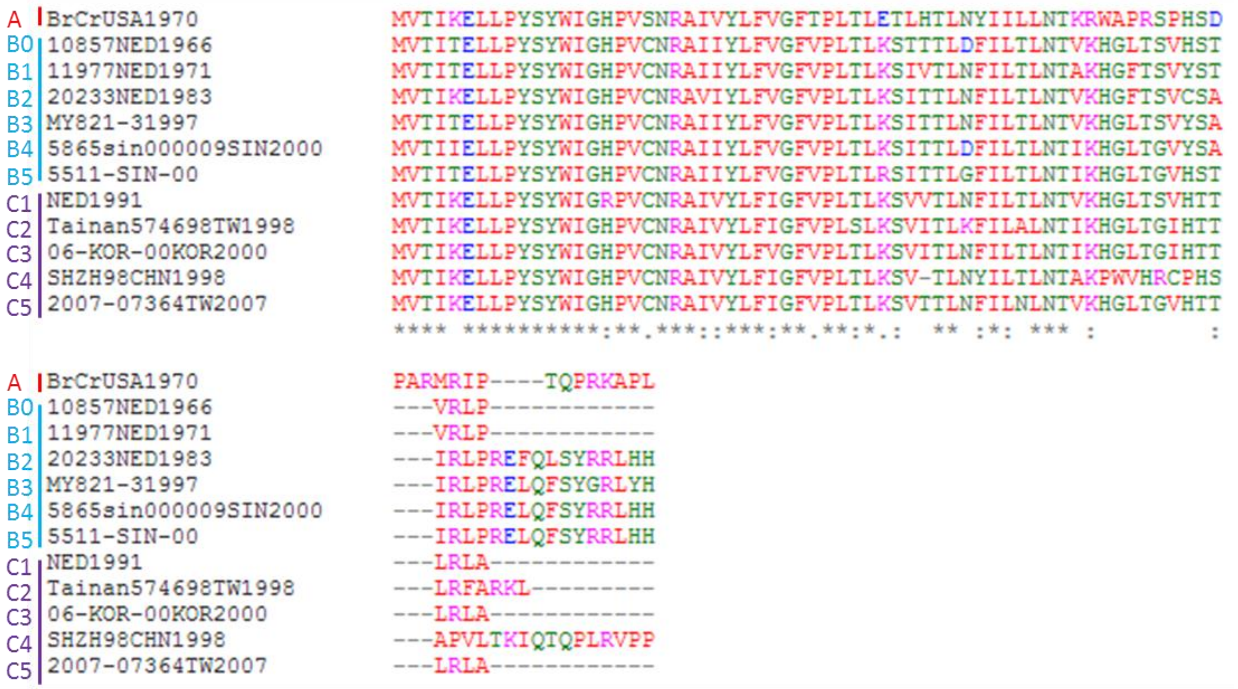

Supplementary Figure 4. Alignment of the amino acid sequences of ORF2p from diverse sub-genotypes of EV-A71.

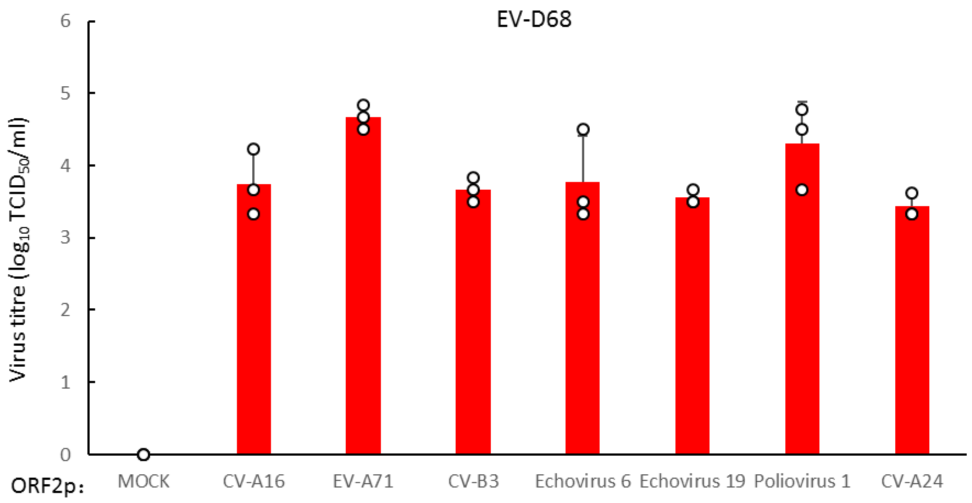

Supplementary Figure 5. Enterovirus ORF2p variants facilitate EV-D68 infection in HT-29 cells. HT-29 cells expressing ORF2p protein derived from CV-A16, EV-A71, CV-B3, Echovirus 6, Echovirus 19, Poliovirus 1, and CV-A24 were infected with EV-D68 at an MOI of 0.5. Viral titres were determined at 72 hpi (n = 3 biologically independent experiments). Error bars denote SEM.

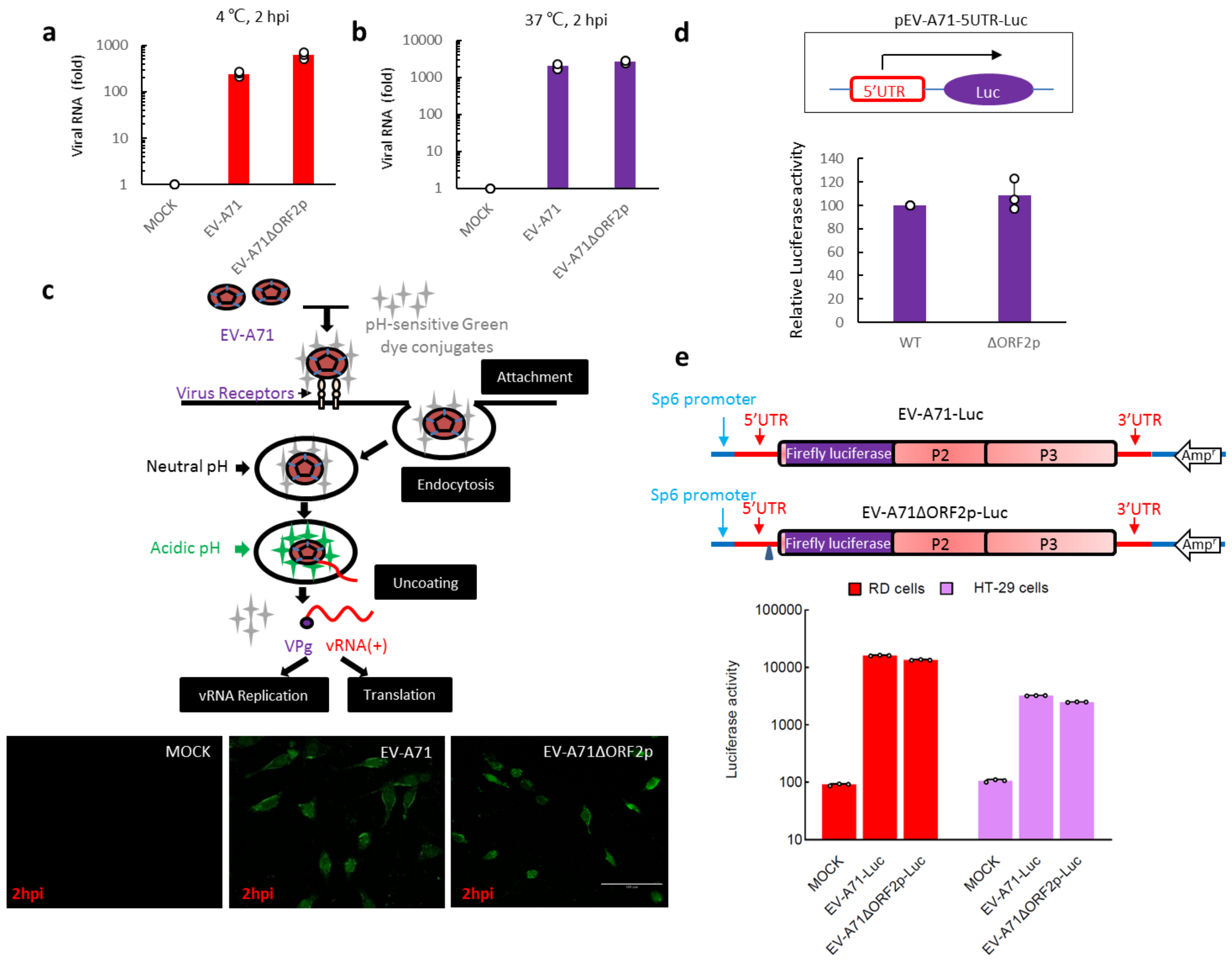

Supplementary Figure 6. ORF2p is dispensable for EV-A71 attachment, endocytosis and 5'UTR activity. a, HT-29 cells were incubated at 4 ℃ for 30 min before infection and then incubated with EV-A71 or EV-A71ΔORF2p at 4 ℃ for 2 h to facilitate virus attachment (n = 3 biologically independent experiments). Error bars denote SEM. b, Cells were infected with EV-D68 and incubated at 37 ℃ for 2 h (n = 3 biologically independent experiments). Error bars denote SEM. qRT-PCR was performed to quantify viral RNA. c, Assay of the acidification of EV-A71-containing endocytic vesicles (top panel). EV-A71 or EV-A71ΔORF2p were labelled with pH-sensitive green dye and then incubated with cells. Fluorescence was detected in live cells at 2 hpi using Alexa Fluor 488 filters and confocal microscopy. Fluorescence increased with the acidity of the surrounding environment. Scale bars equal 100 µm. (bottom panel). d, We generated a firefly luciferase reporter assay to detect activity of the 5′UTR of EV-A71 or EV-A71ΔORF2p. pEV-A71-5UTR-Luc (500 ng) was transfected into HT-29 cells, and luciferase activity was detected at 48 h post-transfection (n = 3 biologically independent experiments). Error bars denote SEM. e, We generated EV-A71 or EV-A71ΔORF2p pseudoviruses with the encapsidated luciferase-encoding replicon (top panel). Pseudoviruses were packaged in HEK293T cells transfected with EV-A71-Luc or EV-A71ΔORF2p-Luc with the EV-A71 P1 expression vector. RD and HT-29 cells were challenged with equal amount of the indicated pseudoviruses and harvested at 9 hpi for luciferase activity assays. Error bars denote SEM, n = 3 biologically independent experiments (bottom panel).

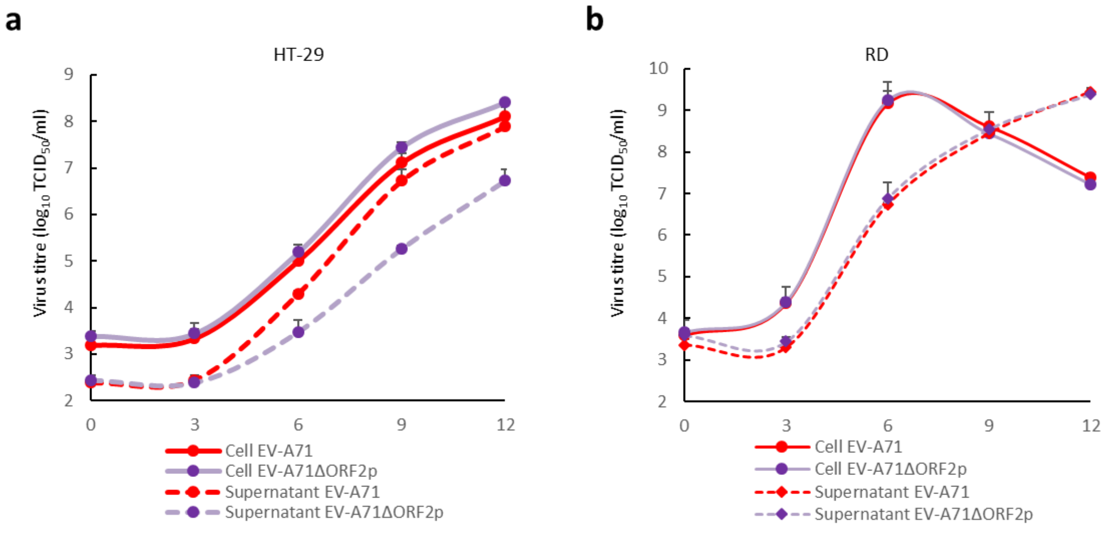

Supplementary Figure 7. One-step growth curves of EV-A71 and EV-A71ΔORF2p viruses. a, b, HT-29 cells (a) and RD cells (b) were challenged with EV-A71 or EV-A71ΔORF2p at an MOI of 10. Virus titres of the supernatant or cell lysates were determined at the indicated times (n = 3 biologically independent experiments). Error bars denote SEM.

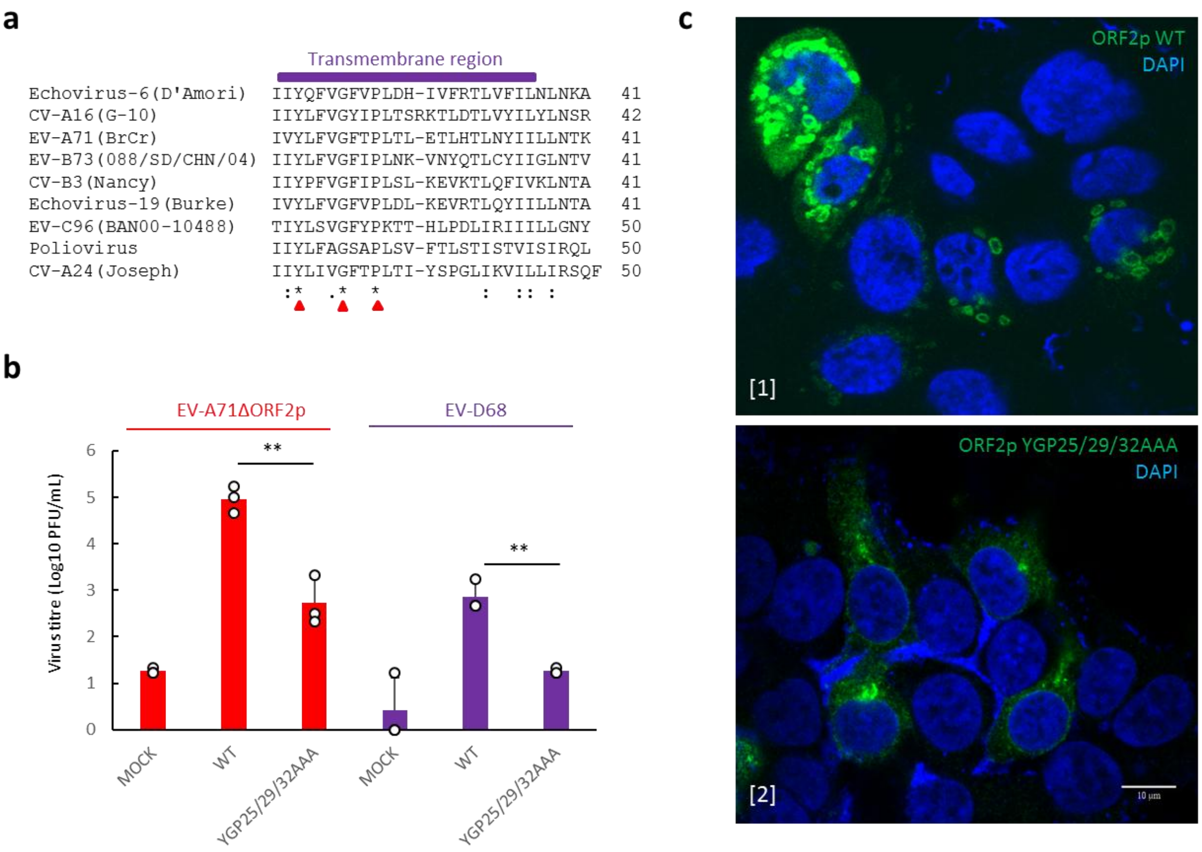

Supplementary Figure 8. Conserved residues in the putative transmembrane region of ORF2p from enteroviruses are critical for ORF2p-mediated virus replication. a, Alignment of amino acid sequences of the predicted transmembrane portion of ORF2p from human enteroviruses. Conserved residues are marked with red arrows. b, HT-29 cells expressing wild-type ORF2p or YGP25/29/32AAA and control cells were infected with EV-A71ΔORF2p or EV-D68. Viral titres were determined at 72 hpi. Error bars denote SEM; ANOVA test, n = 3 biologically independent experiments; $^{**}p < 0.01$. c, subcellular localization of wild-type ORF2p [1] or YGP25/29/32AAA [2]. HT-29 cells transfected with pEV-A71 ORF2p-HA were fixed, permeabilized, and stained with HA-Tag (6E2) Mouse mAb (Alexa Fluor® 488 Conjugate); nuclei were stained with DAPI (4′,6-diamidino-2-phenylindole). The cells were examined by deconvolution microscopy. Scale bars equal 10 µm.

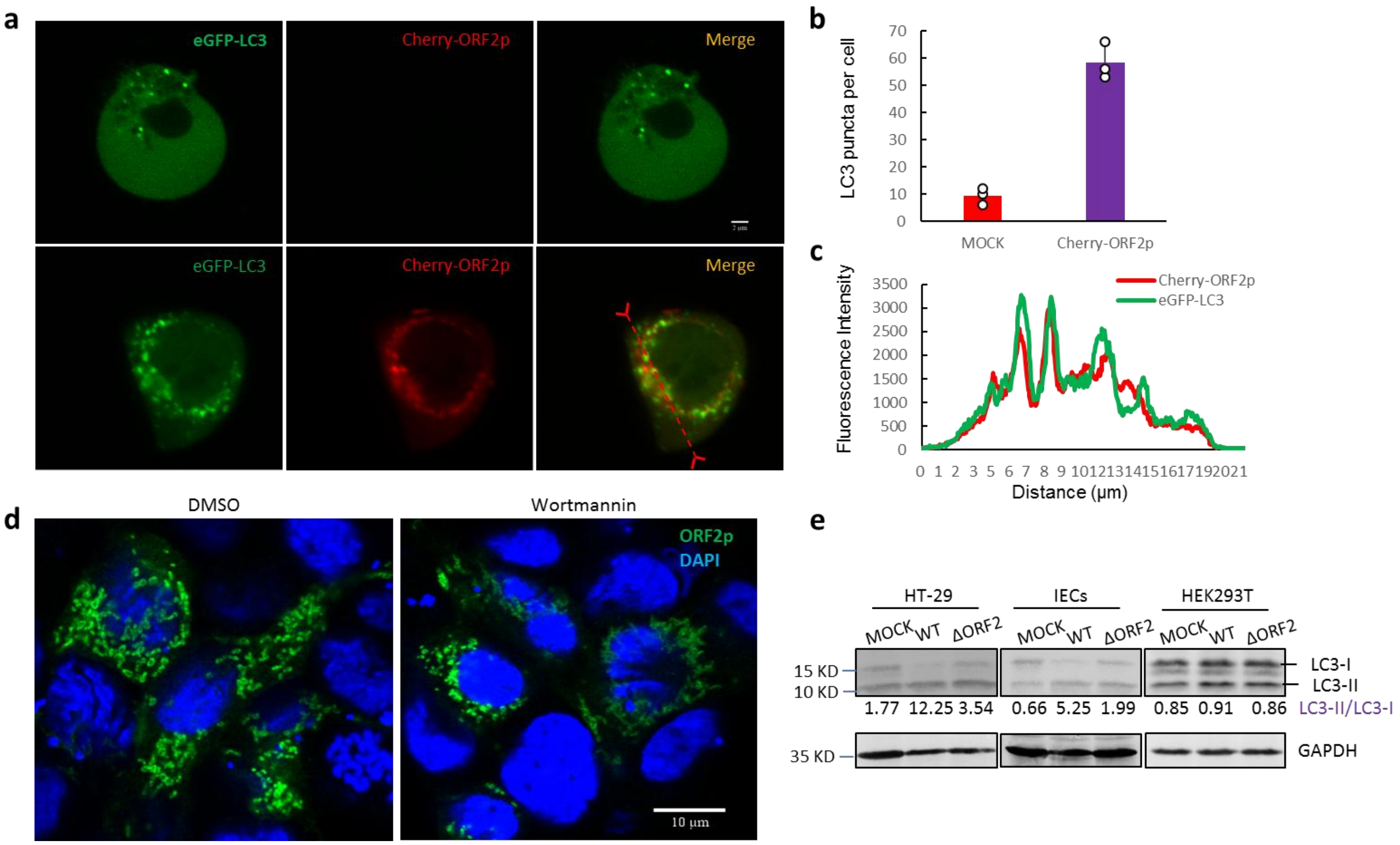

Supplementary Figure 9. EV-A71 ORF2p enhances the autophagic response in human intestinal HT-29 cells. a, Cellular localization of mCherry-tagged ORF2p and eGFP-LC3 proteins in HT-29 cells using live-cell imaging. Scale bars equal 2 μm. b, The numbers of LC3 puncta per cell were quantified using ImageJ software (Error bars denote SEM, 30 cells analyzed per condition). c, Quantitative analysis using ImageJ software of Cherry-ORF2p colocalization with eGFP-LC3 to measure the fluorescence intensity of the indicated region in Fig. 9a (red line). d, HT-29 cells transfected with pEV-A71 ORF2p-HA in the presence of DMSO or wortmannin were fixed, permeabilized, and stained with HA-Tag (6E2) Mouse mAb (Alexa Fluor® 488 Conjugate); nuclei were stained with DAPI. The cells were examined by deconvolution microscopy. Scale bars equal 10 μm. e, HT-29 and HEK293T cells were infected with EV-A71 or EV-A71ΔORF2p at an MOI of 0.1. After 72 h, the cells were harvested for immunoblotting using an anti-LC3B antibody. The numbers under each band represent the intensity of each band measured by densitometric analysis using ImageJ software.

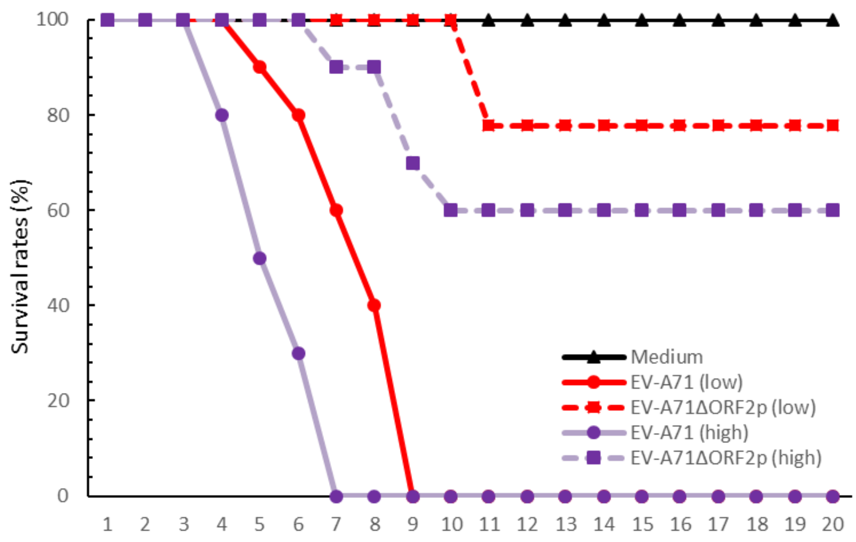

Supplementary Figure 10. Distinct lethality of EV-A71 and EV-A71ΔORF2p viruses in neonatal mice. Newborn ICR mice were infected with the indicated viruses (10^5 $CCID_{50}$/ml or $10^{6.5}$ $CCID_{50}$/ml). Negative control mice were inoculated with DMEM instead of virus. Survival rates and mean clinical scores were continually observed for 20 days after infection.

Supplementary Table 1. Oligonucleotide primers used in this study.

**Primer use**	**Sequence (5'-3')**
Forward primer to introduce EV-A71 mutation OR-HA-F2	CCCAGATTACGCG CTCAAACTCAGCCACTGAG
Reverse primer to introduce EV-A71 mutation OR-HA-F2	ACGTCGTAAGGGTATTTTCGTGAGAG CCGGAG
Forward primer to introduce EV-A71 mutation EV-A71ΔORF2p	GACAATTAAAtAATTGTTACCATATA GCTATTG
Reverse primer to introduce EV-A71 mutation EV-A71ΔORF2p	ACCATAAGCAGCCAGTATAAAG
Forward primer to introduce EV-A71 ORF2p mutation WIG13/15AAA	CTGCCCATCCGGTGTGCAACAG
Reverse primer to introduce EV-A71 ORF2p mutation WIG13/16AAA	CCGCATAGCTATATGGTAACAATTCT TTAATTGTC
Forward primer to introduce EV-A71 ORF2p mutation HPV16/18AAA	CGGCGTGCAACAGAGCAATTGTTTAC
Reverse primer to introduce EV-A71 ORF2p mutation HPV16/18AAA	CAGCGCCAATC CAATAGCTATATG
Forward primer to introduce EV-A71 ORF2p mutation YGP25/29/32AAA	tgctttcgtagCATTAACCTTGAAGTCTGTG
Reverse primer to introduce EV-A71 ORF2p mutation YGP25/29/32AAA	atgaatagggcAACAATTGCTCTGTTGCAC
Forward primer to generate EV-A71 replicon (1, 5'UTR-VP4)	aggtgacactataggTTAAAACAGCCTGTGG GTTGCACC
Reverse primer to generate EV-A71 replicon (1, 5'UTR-VP4)	CTCAGTGGCTGAGTTTGAGTTTTCG
Forward primer to generate EV-A71 replicon (2, Luciferase)	aactcagccactgagATGGAAGACGCCAAAA ACATAAAG
Reverse primer to generate EV-A71 replicon (2, Luciferase)	atttcccCACGGCGATCTTTCCGCCCTTC TT
Forward primer to generate EV-A71 replicon (3, 3'UTR)	gatcgccgtgGGGAAATTTGGACAACAGT CTGGG
Reverse primer to generate EV-A71 replicon (3, 3'UTR)	aaagtgccacctgacgcgtTTTTTTTTTTTTTTT TTTTTTTTTG
Forward primer for RT-PCR (Enterovirus)	ACATGGTGTGAAGAGTCTATTGAGCT
Reverse primer for RT-PCR (Enterovirus)	CCAAAGTAGTCGGTTCCGC
Forward primer for RT-PCR (GAPDH)	GCAAATTCCATGGCACCGT
Reverse primer for RT-PCR (GAPDH)	TCGCCCCACTTGATTTTGG
