# Supplementary material for: A second open reading frame in human enterovirus determines viral replication in intestinal epithelial cells
Source: Nat Commun. 2019 Sep 6;10:4066. doi: 10.1038/s41467-019-12040-9 (PMC6731315; doi:10.1038/s41467-019-12040-9)
Supplement: Supplementary file 3 — Reporting Summary [file 41467_2019_12040_MOESM3_ESM.pdf]

nature research

Corresponding author(s): Wei Wei

Last updated by author(s): Aug 6, 2019

Reporting Summary

Nature Research wishes to improve the reproducibility of the work that we publish. This form provides structure for consistency and transparency in reporting. For further information on Nature Research policies, see Authors & Referees and the Editorial Policy Checklist.

Statistics

For all statistical analyses, confirm that the following items are present in the figure legend, table legend, main text, or Methods section.

n/a	Confirmed	
☐	☒	The exact sample size (*n*) for each experimental group/condition, given as a discrete number and unit of measurement
☐	☒	A statement on whether measurements were taken from distinct samples or whether the same sample was measured repeatedly
☐	☒	The statistical test(s) used AND whether they are one- or two-sided<br>*Only common tests should be described solely by name; describe more complex techniques in the Methods section.*
☐	☒	A description of all covariates tested
☐	☒	A description of any assumptions or corrections, such as tests of normality and adjustment for multiple comparisons
☐	☒	A full description of the statistical parameters including central tendency (e.g. means) or other basic estimates (e.g. regression coefficient) AND variation (e.g. standard deviation) or associated estimates of uncertainty (e.g. confidence intervals)
☐	☒	For null hypothesis testing, the test statistic (e.g. *F*, *t*, *r*) with confidence intervals, effect sizes, degrees of freedom and *P* value noted<br>*Give P values as exact values whenever suitable.*
☒	☐	For Bayesian analysis, information on the choice of priors and Markov chain Monte Carlo settings
☒	☐	For hierarchical and complex designs, identification of the appropriate level for tests and full reporting of outcomes
☒	☐	Estimates of effect sizes (e.g. Cohen's *d*, Pearson's *r*), indicating how they were calculated

Our web collection on statistics for biologists contains articles on many of the points above.

Software and code

Policy information about availability of computer code

Data collection	cellSens.
Data analysis	DNAMAN,Clustal Omega, Stata.

For manuscripts utilizing custom algorithms or software that are central to the research but not yet described in published literature, software must be made available to editors/reviewers. We strongly encourage code deposition in a community repository (e.g. GitHub). See the Nature Research guidelines for submitting code & software for further information.

Data

Policy information about availability of data

All manuscripts must include a data availability statement. This statement should provide the following information, where applicable:

- Accession codes, unique identifiers, or web links for publicly available datasets
- A list of figures that have associated raw data
- A description of any restrictions on data availability

All data presented herein is available both in raw and in processed format. There are no restrictions to this statement.

Field-specific reporting

Please select the one below that is the best fit for your research. If you are not sure, read the appropriate sections before making your selection.

☒ Life sciences ☐ Behavioural & social sciences ☐ Ecological, evolutionary & environmental sciences

For a reference copy of the document with all sections, see nature.com/documents/nr-reporting-summary-flat.pdf

Life sciences study design

All studies must disclose on these points even when the disclosure is negative.

Sample size	No statistical methods were used to predetermine the sample sizes.
Data exclusions	No data were excluded.
Replication	The replications were successful. All experiments were performed in three or more biological replicates.
Randomization	No method of randomization was used.
Blinding	The investigators have not been blinded during data collection and analysis.

Reporting for specific materials, systems and methods

We require information from authors about some types of materials, experimental systems and methods used in many studies. Here, indicate whether each material, system or method listed is relevant to your study. If you are not sure if a list item applies to your research, read the appropriate section before selecting a response.

Materials & experimental systems

n/a	Involved in the study	
☐	☒	Antibodies
☐	☒	Eukaryotic cell lines
☒	☐	Palaeontology
☐	☒	Animals and other organisms
☒	☐	Human research participants
☒	☐	Clinical data

Methods

n/a	Involved in the study	
☒	☐	ChIP-seq
☒	☐	Flow cytometry
☒	☐	MRI-based neuroimaging

Antibodies

Antibodies used	The antiserum was generated by immunizing rabbits with a polypeptide comprising the C-terminal 20 residues of ORF2, and the antibody was purified by using an ORF2 antigen column (HuaBio, Hangzhou,China). An anti-Enterovirus 71 VP1 antibody (GTX132338) and an anti-Enterovirus D68 VP1 antibody (GTX132313) were purchased from GeneTex (San Antonio, USA). The monoclonal mouse anti-α-tubulin antibody (A01410) was purchased from GenScript (Piscataway, USA). A polyclonal rabbit anti-HA antibody (71-5500) was purchased from Thermo Fisher Scientific (Carlsbad, USA). HA-Tag (6E2) Mouse mAb (Alexa Fluor® 488 Conjugate)(2350) was from Cell Signaling Technology, Inc. (Minnesota, USA).An anti-LC3B antibody (L7543) was purchased from Sigma (Darmstadt, Germany).
Validation	For antibodies obtained from commercial sources listed in the above section, validation was performed by the vendors.

Eukaryotic cell lines

Policy information about cell lines

Cell line source(s)	HT-29 human IECs (Cell Bank of the Chinese Academy of Sciences, TCHu103), LS 174T human IECs (ATCC, CL-188), 293T human embryonic kidney cells (ATCC, CRL-3216), RD human rhabdomyosarcoma cells (ATCC, CCL-136), HeLa human cervical epithelial cells (ATCC, CCL-2), Vero African green monkey kidney cells (ATCC, CCL-81) , HepG2 human hepatocellular carcinoma cells (ATCC, HB-8065), A549 (ATCC, CRM-CCL-185), NSC-34 mouse motor neuron cells (Cedarlane Laboratories, CLU140), U937 human lymphoma cells (ATCC, CRL-1593.2), HCT-8 human IECs (ATCC, CCL-244), Hce-8693 human IECs (Cell Bank of the Chinese Academy of Sciences, TCHu 70) , LS 513 human IECs (Cell Bank of the Chinese Academy of Sciences, TCHu237) , LoVo human IECs (Cell Bank of the Chinese Academy of Sciences, TCHu 82) , RKO human IECs (ATCC, CRL-2577) .
Authentication	None as directly purchased from ATCC, Cedarlane Laboratories, or Cell Bank of the Chinese Academy of Sciences.
Mycoplasma contamination	Cell lines were routinely tested for mycoplasma and immediately tested upon suspicion. None of the lines used in the reported experiments tested positive.
Commonly misidentified lines (See ICLAC register)	None of the cell lines used are listed in the ICLAC database.

Animals and other organisms

Policy information about studies involving animals; ARRIVE guidelines recommended for reporting animal research

Laboratory animals	One-day-old specific-pathogen-free (SPF) ICR neonatal mice (Experimental Animal Center, Jilin University) were used to establish the animal model of viral infection. Both male and female animals were used in the experiments.
Wild animals	No wild animals were used in the study.
Field-collected samples	No field-collected samples were used in the study.
Ethics oversight	All animal experiments were conducted according to animal protocols approved by the Insititute of Virology and AIDS Research Subcommittee of Research Animal Care.

Note that full information on the approval of the study protocol must also be provided in the manuscript.
